# Factors associated with death in hospitalized pneumonia patients with 2009 H1N1 influenza in Shenyang, China

**DOI:** 10.1186/1471-2334-10-145

**Published:** 2010-05-31

**Authors:** Wei Cui, Hongwen Zhao, Xu Lu, Ying Wen, Ying Zhou, Baocheng Deng, Yu Wang, Wen Wang, Jian Kang, Pei Liu

**Affiliations:** 1 Department of Infectious Diseases, the First Affiliated Hospital, China Medical University, Shenyang, China; 2 Department of Respiratory Medicine, the First Affiliated Hospital, China Medical University, Shenyang, China

## Abstract

**Background:**

During the spring of 2009, a pandemic influenza A (H1N1) virus emerged and spread globally. We describe the clinical characteristics and factors associated with the death of patients who were hospitalized with 2009 H1N1 influenza pneumonia in Shenyang, China, from November to December 2009.

**Methods:**

We carried out a retrospective chart review of 68 patients who were hospitalized with pneumonia and confirmed to have 2009 H1N1 virus infection by a real time RT-PCR assay of respiratory specimens.

**Results:**

Of the 68 patients we studied, 30 (44%) were admitted to an intensive care unit and 10 (14.7%) died. The median age of patients was 41 years (range, 18-66), and only one patient was over 65 years of age. The male to female ratio was 2.78:1 (50:18). Of the 68 patients, 23 (34%) had at least one underlying medical condition, 9 (13%) had a cigarette index ≥400 and 22 (32%) were obese. All patients underwent chest radiography on admission and the findings were consistent with pneumonia in all cases. All patients were treated with oseltamivir and treatment was initiated at a median time of seven days after the onset of illness. The laboratory test results indicated lymphopenia, hypoproteinemia and elevated lactic dehydrogenase and C reactive protein levels. Of the 68 patients, 33 (52%) showed a reduction in CD4 T cell counts. Of the 58 patients who survived, 31 (53%) had lymphopenia and 27 recovered from this condition after five days. Of the 10 patients who died, nine (90%) had lymphopenia and only two patients recovered from this condition after five days. Obesity and recovery from lymphopenia after five days were factors associated with death, as determined by multivariate logistic-regression analysis (obesity, odds ratio = 23.06; lymphocytopenia reversion, odds ration = 28.69).

**Conclusions:**

During the evaluation period in Shenyang, China, 2009 H1N1 influenza caused severe illness requiring hospitalization in 68 patients, 10 (14.7%) of which died. Many of these patients were considered healthy adults and few were elderly (65 years or older). Obesity and lymphopenia, which was not restored after five days of treatment, were factors associated with poor outcomes of 2009 H1N1 influenza infection.

## Background

In early April 2009, cases of human infection with 2009 pandemic influenza A (H1N1) virus were identified in the United States [1,2] and Mexico [3], and the virus then spread rapidly to other regions of the world [4,5]. On December 20, 2009, more than 208 countries and overseas territories or communities had reported laboratory confirmed cases of pandemic influenza A (H1N1), and at least 11,516 deaths had occurred [6]. The first case of 2009 pandemic influenza A (H1N1) virus infection in China was documented on May 10. From May to November of 2009, the number of reported cases in China was 69,160 [China Centre for Disease Control, CDC]. The majority of the early cases reported mild, influenza-like illnesses [7-11], but more severe infections have been reported as the pandemic spreads and the number of infected individuals increases. The greatest number of cases of 2009 H1N1 influenza infection resulting in severe clinical presentations and death have been reported in Mexico and other Western countries. The fatality rate of hospitalized patients with critical illness due to 2009 H1N1 influenza was about 10% [12-14] in United States and Mexico. The clinical features included fever and respiratory symptoms and death most commonly resulted from severe acute respiratory distress syndrome (ARDS) and refractory hypoxemia [7,15-18]. Obesity, underlying health conditions and delayed neuraminidase inhibitor treatment were the major risk factors for a poor outcome of infection [17,18]. However, the clinical outcomes and risk factors associated with 2009 H1N1 influenza infection in China remain to be determined and variables that could help clinicians to identify patients at high risk of infection would be valuable. Therefore, this report summarizes the clinical manifestations, clinical outcomes and the risk factors associated with death in hospitalized pneumonia patients associated with 2009 H1N1 influenza infections in Shenyang, China, during November to December 2009.

## Methods

### Patients

We carried out a retrospective chart review of 68 patients who were hospitalized with pneumonia and 2009 H1N1 influenza infection in the First Affiliated Hospital of China Medical University from November 4, 2009, to December 31, 2009. During the 2009 H1N1 influenza epidemic, patients in clinics or emergency departments who presented with an influenza-like illness were tested for 2009 H1N1 influenza virus, and only those patients whose illness was complicated with pneumonia were admitted to our department. Patients with mild symptoms were isolated for treatment at home. Therefore, in total 68 patients were treated in our department between November 4^th ^and December 31^st^. We retrospectively studied these 68 cases without any selection. The First Affiliated Hospital of China Medical University is a tertiary care center that includes obstetric services, pediatric wards and oncology wards, and treats immunosuppressed patients and HIV-infected patients. It is also a tertiary referral center for adult patients infected with 2009 H1N1 influenza, as appointed by the national government. However, this hospital is not a tertiary referral center for children and pregnant women infected with 2009 H1N1 influenza, so no children and pregnant women were involved in our study. All patients were confirmed positive for 2009 H1N1 influenza A by a real-time reverse-transcriptase-polymerase-chain-reaction assay on respiratory specimens carried out at the CDC. All tests used standard CDC-based primers. All patients underwent chest radiography on admission. The study was approved by the Ethics Committee of China Medical University.

### Study Design

Medical-chart abstractions were performed by physicians from the Infectious Diseases Department. They used a standardized form that included demographic data, seasonal influenza vaccination history for the previous year, smoking status, underlying medical conditions, clinical signs and symptoms, selected laboratory tests including C reactive protein (CRP), white blood cell classification and count, lactic dehydrogenase (LDH), creatine kinase (CK), glutamic-oxaloacetic transaminase (AST), glutamic alanine aminotransferase (ALT), albumin (Alb), CD4, CD8 and CD3 T cell counts, blood gas analyses, blood or sputum cultures, radiographic findings, intervals between symptom onset and initiation of oseltamivir therapy, treatment course and length of stay. The thresholds for each laboratory test are listed in annex and any elevation or reduction according to the threshold of normal was defined as an abnormality. The body mass index (BMI) (the weight in kilograms divided by the square of the height in meters) of each patient was calculated. Patients were defined as overweight if their BMI was 24 to 27.9 and obese if their BMI ≥28 according to the criterion established by the Working Group On Obesity In China (WGOC) in 2005. Lymphocytopenia was defined as an absolute lymphocyte count ≤800 cells/μL. A cigarette index abnormality was defined as ≥400. Blood gas analyses were tested by Nova Biomedical Stat Profile, and the oxygenation index (PaO_2_/FiO_2_) was calculated and abnormality was defined as values ≤300. Blood cultures were tested using the BactecTM 9240 System (Becton and Dickinson Company).

### Statistical Analysis

Continuous variables were summarized as the mean values (±SD). For categorical variables, the percentage of patients in each category was calculated. Clinical characteristics were compared between subgroups of survivors and deceased and between patients who were admitted to an intensive care unit (ICU) and those who were not, with the use of a non-parametric Mann-Whitney U test, chi-square test or Fisher's exact test, as appropriate. A P value of less than 0.05 was considered to indicate statistical significance. We performed bivariate analysis to compare the clinical characteristics of patients who survived with those of patients who died. We used multivariate logistic-regression models to further investigate associations between illness and mortality. Data with a P value < 0.05 was entered into the multivariate logistic regression model and data with a P value < 0.1 was kept in the model. All analyses were carried out with the use of SPSS software for Windows (release 13.0) (from http://www.bizinsight.com.cn).

## Results

### Clinical Characteristics

This report describes the 68 patients hospitalized in the First Affiliated Hospital of China Medical University with pneumonia and 2009 H1N1 virus infection from November 4 to December 31, 2009. The median age of the patients was 41 years (range, 18-66 years). 67 (99%) of patients were 18-59 years of age with only one (1%) patient over 65 years of age. The male to female ratio was 2.78:1 (50:18). Of the 68 patients, 23 (34%) had an underlying medical condition and five (7%) had at least two such conditions. These conditions included diabetes, cardiovascular, hepatic, renal, neurologic and pulmonary diseases (Table 1). Nine (13%) patients had a cigarette index ≥400. Height and weight measurements were available for all 68 patients. 20 (29%) of the patients were overweight and 22 (32%) were obese (Table 1). None of the patients had been vaccinated for seasonal or 2009 H1N1 influenza during the 2008-2009 season (an appointed population, including doctors, were vaccinated for 2009 H1N1 influenza in October, whereas other members of the population were vaccinated for after December, 2009, in Shenyang). Symptoms at presentation included a fever, cough, difficulty breathing, hemosputum, diarrhea and myalgia. All patients presented with a fever. A cough was reported in 60 (88%) patients, breathing difficulties were reported in 31 (46%) patients, hemosputum in 11 (16%) patients, diarrhea in 5 (7%) patients and myalgia in 18 (27%) patients. The median duration of symptoms before hospitalization was seven days (range, 5-20 days).

**Table 1:** Baseline characteristics of hospitalized patients with pneumonia and 2009 H1N1 influenza in the First Affiliated Hospital of China Medical University during November 4 to December 31, 2009.

Characteristic	No. (%)
Female sex	18 (26)
Age group:	
18-49	56 (82)
50-64	11 (16)
≥ 65	1 (1)
Medical condition:	
Any one condition	23 (34)
Asthma	1
Chronic obstructive pulmonary disease	3
Tuberculosis	2
Diabetes	2
Congenital heart disease	1
Rheumatic heart disease	2
Hypertension	9
Chronic renal failure	1
Nephritic syndrome	1
Kidney transplant	1
Chronic hepatitis type b	1
Liver cirrhosis	1
Fatty liver disease	1
Guillain-Barre syndrome	1
Pituitary adenoma	1
Cigarette index ≥ 400	9 (13)
BMI:	
24-27.9	20 (29)
≥ 28	22 (32)

### Diagnostic Findings

Five patients showed baseline chronic abnormalities in the results of laboratory tests as a result of their underlying medical conditions. Three patients showed a reduced level of serum Alb of 31, 27 and 24.5 g/L, one patient showed a mild elevation of ALT at 67 U/L and one deceased patient showed an elevated level of Cr at 340 μmol/L. The majority of the laboratory test data was acquired within 48 h of hospital admission, including CRP, LDH, CK, AST, ALT, Alb, CD4, CD8 and CD3 T cell count and blood gas analyses. As for white blood cell classification and counts, there were 59 patients whose samples were taken in the clinics or emergency rooms of our hospital and nine patients whose samples were taken in other hospitals. The median interval from the onset of illness to the time of sample collection was five days (range, 3-9). Ten patients ultimately died and the median interval from the time of sample collection to death was seven days (range, 5-21). The lymphocyte counts for all patients were monitored every other day until their lymphocyte counts recovered to normal levels. Laboratory tests abnormalities included lymphopenia (59%), elevated serum LDH (84%), CK (37%), AST (56%), ALT (38%) and CRP (63%). All patients were tested for HIV infection and were negative. Of the 64 patients, 33 (52%) had reduced CD4 T cell counts.

Blood cultures were performed for patients who displayed a high fever above 38.0°C for at least three days or patients who had a repeated fever. Sputum cultures were also performed for patients who showed symptoms of expectoration especially in cases where the sputum was yellowish and purulent. All but three patients had received antibiotics prior to sample collection. Five of 11 patients had positive blood cultures: two of which were *Acinetobacter baumannii*, one was methicillin-resistant *Staphylococcus cohnii*, one was methicillin-resistant *Staphylococcus saprophyticus *and one was methicillin-resistant *Staphylococcus aureus*. Nine of 29 patients had positive sputum cultures: six of which were *A. baumannii*, two were *Pseudomonas aeruginosa *and one was methicillin-resistant *S. aureus*. All 68 patients who underwent chest radiography on admission displayed clinical signs that were consistent with pneumonia. Radiographic findings included bilateral infiltrates (in 64 patients), an infiltrate limited to one lobe and multi-lobe infiltrates limited to one lung. Of 68 patients who underwent blood gas analysis, 38 (56%) had an oxygenation index below 300.

### Treatment

All 68 patients received oseltamivir treatment. The median time from the onset of illness to the initiation of antiviral therapy was seven days (range, 2-15). Of 68 patients, 50 (74%) were treated with antiviral therapy within 48 hours of hospital admission, and 18 (26%) were treated after 48 hours. All 68 patients received antibiotic therapy, with 78% of the patients receiving more than one antibiotic. The median value for the number of antibiotics administered was two (range, 1-7) and the median duration of antibiotic treatment was seven days. Commonly used antibiotics included cefminox and levofloxacin. Of the 68 patients, 66 (97%) received corticosteroids by means of an injection. The average dosage of methylprednisolone was 1 mg·kg^-1^·d^-1 ^and the median time for initiation of methylprednisolone in hospital was day 7. Of the 68 patients, 48 (71%) received extrasin alpha1.

### ICU Admissions

Of the 68 patients we evaluated, 30 (44%) were admitted to an ICU and 10 of these died. There were no significant differences in the average age, cigarette index ≥400 and interval time from the onset of illness to the initiation of antiviral therapy between patients who were admitted to an ICU and patients who were not (Table 2). Patients who were admitted to an ICU were more likely than patients who were not admitted to an ICU to have an underlying medical condition and a BMI ≥28 (Table 2). Of the 30 patients who were admitted to an ICU, 16 (53%) had at least one underlying medical condition, including tuberculosis (1), asthma (1), chronic obstructive pulmonary disease (COPD) (1), chronic renal failure (1), chronic hepatitis B virus infection (1), fatty liver disease (1), hypertension (7), congenital heart disease (1), rheumatic heart disease (2), Guillain-Barre syndrome (1) and pituitary adenoma (1). Seven of the 38 (18%) patients who were not admitted to an ICU had an underlying medical condition, including tuberculosis (1), COPD (2), liver cirrosis (1), diabetes (2), hypertension (2), nephrotic syndrome (1) and a kidney transplant (1).

**Table 2:** Comparison between the clinical characteristics and laboratory test abnormalities of patients with pneumonia and 2009 H1N1 influenza who were admitted to an ICU and patients who were not admitted to an ICU in the First Affiliated Hospital of China Medical University during November 4 to December 31, 2009.

Clinical characteristic or laboratory test abnormality	ICU		Non-ICU		P
		
	data	n	data	n	
Medical Condition (no. (%))	16 (53)	30	7 (18)	38	0.003^#^
Cigarette index ≥ 400(no. (%))	5 (17)	30	4 (10)	38	0.458
BMI ≥ 24 (no. (%))	23(77)	30	19(50)	38	0.025^#^
BMI 24-27.9	10 (33)	30	10 (32)	38	0.528
BMI ≥ 28	13 (43)	30	9 (24)	38	0.085
Median age	41 (18-63)	30	41 (18-66)	38	0.990
Interval from onset of illness to initiation of antiviral therapy, in days	7 (4-12)	30	7 (2-15)	38	0.551
Lymphcyto count (×10^9^/L)	0.58 (0.02-4.34)	30	1.065 (0.31-2.48)	38	<0.0005**
LDH (U/L)	1097.5 (218-4340)	30	688 (180-2250)	37	0.005**
CK (U/L)	220 (23-4188)	30	161 (20-2712)	37	0.313
AST (U/L)	66 (12-229)	30	41.5 (13-339)	38	0.107
ALT (U/L)	45.5 (15-188)	30	43.5 (11-204)	38	0.537
Alb (g/L)	28.95 (18-42)	30	37 (9.6-51)	38	<0.0005**
CRP (mg/mL)	2.66 (0.25-29.7)	27	1.23 (0.1-10.2)	38	0.024**
CD4 T cell count (/μL)	232 (29-1892)	28	651 (128-1607)	36	<0.0005**
CD8 T cell count (/μL)	166.5 (23-1109)	28	406 (85-1113)	36	<0.0005**
CD3 T cell count (/μL)	465 (57-3046)	28	1148.5 (216-2496)	36	<0.0005**

**..... Statistically significant with the use of a non-parametric Mann-Whitney U test; ^#^..... Statistically significant with the use of a chi-square test or Fisher's exact test test

BMI: Body Mass Index; LDH: lactic dehydrogenase; CK: creatine kinase; AST: glutamic-oxaloacetic transaminase; ALT: glutamic alanine aminotransferase; Alb: albumin; CRP: C reactive protein.

Laboratory test data showed that the levels of serum LDH and CRP were higher in patients who were admitted to an ICU than those who were not admitted (P < 0.05) (Table 2). Patients who were admitted to an ICU also showed a more significant reduction in serum Alb, lymphocyte counts, CD4, CD8 and CD3 T cell counts, compared with patients who were not admitted to an ICU (P < 0.05) (Table 2). In a multivariable model that included BMI ≥28, medical conditions, LDH, CRP and CD4 T cell count, no variable was significantly associated with admission to an ICU.

Of the 30 patients who were admitted to an ICU, 13 required mechanical ventilation, 8 had acute respiratory distress syndrome (ARDS), and 21 had a clinical diagnosis of sepsis. All patients received antiviral drugs, antibiotics, corticosteroids and extrasin alpha1. The median time from the onset of illness to the initiation of antiviral therapy was seven days (range, 4-12).

### Outcomes

Of the 68 hospitalized patients, 58 (85.3%) were discharged and 10 (14.7%) died. The median time from the onset of illness to death was 11 days (range, 7-26). The median time from hospital admission to death was six days (range, 3-21). The median length of hospital stay for survivors was 11 days (range, 3-37). There were no significant differences in median age, cigarette index ≥400 and interval time from the onset of illness to the initiation of antiviral therapy, between patients who died and patients who survived (Table 3). Patients who died were more likely than patients who survived to have underlying medical conditions and a BMI value ≥28 (Table 3). Of the 10 patients who died, six had at least one underlying medical condition, including tuberculosis (1), chronic renal failure (1), chronic hepatitis B virus infection (1), asthma (1), COPD (1), hypertension (2) and congenital heart disease (1). Of the 58 patients who survived, 17 had an underlying medical condition, including tuberculosis (1), hypertension (7), rheumatic heart disease (2), Guillain-Barre syndrome (1), fatty liver disease (1), pituitary adenoma (1), COPD (2), liver cirrosis (1), diabetes (2), nephrotic syndrome (1) and a kidney transplant (1).

**Table 3:** Comparison of the clinical features and laboratory test abnormalities between patients with pneumonia and 2009 H1N1 influenza who died and those who survived in the First Affiliated Hospital of China Medical University during November 4 to December 31, 2009.

Clinical characteristic or laboratory test abnormalities	Died		Survived		P
		
	data	n	data	n	
Medical condition (no)	6	10	17	58	0.076
Cigarette index ≥ 400(no)	2	10	7	58	0.611
BMI ≥ 24(no)	9	10	33	58	0.045^#^
BMI 24-27.9	1	10	19	58	0.260
BMI ≥ 28	8	10	14	58	0.001^#^
Median age	43.5(24-63)	10	41(18-66)	58	0.562
Interval from onset of illness to initiation of antiviral therapy, in days	6(4-10)	10	7(2-15)	58	0.345
Lymphocyte count (×10^9^/L)	0.58(0.02-1.15)	10	0.79(0.31-4.34)	58	0.025**
LDH(U/L)	2583(324-4340)	10	775(180-4104)	57	0.003**
CK(U/L)	299.5(25-2000)	10	165(20-4188)	57	0.268
AST(U/L)	84(16-225)	10	43(12-339)	58	0.091
ALT(U/L)	54(23-167)	10	41.5(11-204)	58	0.446
Alb(g/L)	27.3(18-33)	10	35(9.6-51)	58	0.003**
CRP (mg/mL)	9.515(2.32-27.1)	8	1.49(0.1-29.7)	57	0.001**
CD4 T cell count (/μL)	118.5(29-391)	8	470.5(86-1892)	56	<0.0005**
CD8 T cell count (/μL)	87(23-293)	8	307(44-1113)	56	<0.0005**
CD3 T cell count (/μL)	219(57-697)	8	784(169-3046)	56	<0.0005**
Lymphocyte count ≤ 0.8×10^9^/L (no)	9	10	31	58	
Duration of lymphocytopenia	5.5 (4-19)	10	5 (3-9)	58	
Duration of lymphocytopenia > 5 days (no)	7	10	4	58	

**..... Statistically significant with the use of a non-parametric Mann-Whitney U test; ^#^..... Statistically significant with the use of a chi-square test or Fisher's exact test test

BMI: Body Mass Index; LDH: lactic dehydrogenase; CK: creatine kinase; AST: glutamic-oxaloacetic transaminase; ALT: glutamic alanine aminotransferase; Alb: albumin; CRP: C reactive protein.

Laboratory test results were compared between patients who survived and those who died. No differences were found in the levels of serum CK, AST and ALT between patients who survived and those who died. However, patients who died were more likely than patients who survived to have elevated levels of serum LDH and CRP and reduced levels of Alb and CD4, CD8 and CD3 T cell counts (P < 0.05) (Table 3). In addition, we noticed that 40 patients suffered from lymphocytopenia during the early stage of illness. Among these patients, 31 recovered and nine died. Of the 31 surviving patients, 27 (87%) recovered from lymphocytopenia within five days, while just two of the patients who later died recovered from lymphocytopenia within five days (Table 3).

In a multivariate model that included BMI ≥28, medical conditions, CRP, CD4 T cell counts and lymphocytopenia reversion, the variables that were significantly associated with death were BMI ≥28 and delayed lymphocytopenia recovery (Table 4). All patients who died had received antiviral drugs, antibiotics and corticosteroids. The median time from the onset of illness to the initiation of antiviral therapy was six days (range, 4-10).

**Table 4:** Results of multivariate logistic analysis to compare the outcomes of patients who survived with those who died.

variables entered into the model	Variables in the equation	B	S.E.	OR	P	95% CI
BMI ≥ 28, medical conditions CRP	BMI ≥ 28	3.138	1.468	23.060	0.033	1.298-409.769
CD4 T cell counts lymphocytopenia reversion	Lymphocytopenia reversion	3.356	1.294	28.687	0.01	2.270-362.507

## Discussion

Here, we report on a series of hospitalized patients with pneumonia caused by 2009 H1N1 influenza infection in Shenyang, China, from November to December 2009. During the period of our study, the pandemic strain of H1N1 virus caused severe illness, including pneumonia and ARDS, and resulted in ICU admissions in 44% of patients and death in 14.7% of patients. These findings were similar to the majority of reports in other countries [14,17,19,20].

In our study, almost 80% of the hospitalizations due to H1N1 influenza infection involved individuals who were between the ages of 18 and 49 years. The male to female ratio was 2.78:1. These age and sex distributions were different from those reported nationally for the normal population in China (male to female ratio was 106.30:100; age distributions were: 15-59 years, 68.70%; 60 years and over, 11.03%; 65 years and over, 7.69%). We concluded that severe illness resulting from H1N1 virus infection was more likely among male adults in Shenyang, which was consistent with other reports [19,21-25]. Persons aged 50 years or older showed a low incidence of 2009 H1N1 influenza infection, this may be due to "protection" by preexisting immunity resulting from previous exposure to H1N1 influenza infection, along with reduced outdoor activity by these individuals because of the fear of H1N1 infection. A recent study demonstrated that persons aged 50 years or older who were hospitalized with pandemic 2009 influenza A (H1N1) infection were among those most likely to die, despite having lower hospitalization rates [14]. However, our findings did not support this conclusion, possibly due to the small sample size used in our study. Nevertheless, it is appropriate that clinicians should closely monitor elderly patients with pandemic 2009 H1N1 influenza infection and treat them accordingly.

In our study, almost 30% of patients had pre-existing medical conditions, of which chronic pulmonary diseases, diabetes and hypertension were the most common diseases, as found in other studies [19,24,25]. The patients included in our study who died had a higher rate of pre-existing diseases. Asthma is also considered a high risk factor for 2009 H1N1 influenza infection, however, our findings did not confirm this as only one patient in our study had asthma as a pre-existing condition. This discrepancy might be caused by the lower morbidity of asthma in China (approximately 2%) compared with Western counties (approximately 10-30%). COPD and heart disease are also considered high risk factors for 2009 H1N1 influenza infection, and in our study about two thirds of the patients with pre-existing medical conditions suffered from COPD or heart disease. Thus, it is appropriate that physicians should pay close attention to patients with COPD or chronic heart disease during the 2009 H1N1 influenza epidemic.

In our study, 32% of patients were obese compared with 7% of adults in the normal population in China, which indicated severe illness from H1N1 virus infection was more likely among obese individuals, as reported in other studies [17,18,24,26,27]. Almost 80% of patients who died from H1N1 influenza infection were obese and using multivariate logistic-regression models, obesity was found to be a factor associated with death from 2009 H1N1 influenza in our study, despite the wide 95% confidence interval (CI) for the odds ratio (OR) for BMI due to the limitations of using a small sample size.

The clinical features of patients who were hospitalized with 2009 H1N1 influenza infection included fever, cough, myalgia and dyspnea, which were generally similar to other reports. Whereas the incidence of gastrointestinal symptoms such as nausea, vomiting, and diarrhea was much lower than previously reported [7,15-18,28,29]. The results of laboratory tests indicated lymphopenia, hypoproteinemia, elevated LDH and CRP levels, which were consistent with other reports [19,29,30]. Abnormalities in the laboratory test results were more significant in patients who were admitted to an ICU and/or died than in patients who were not admitted to an ICU, but these abnormalities were not predictive factors in ICU admission or death. Our results were not consistent with the study in Taiwan which found initial lymphocyte count less 800/microL was associated with the development of respiratory failure [31]. Although lymphopenia was not a risk factor for death in our study, we found lymphopenia was restored after about five days in most surviving patients, whereas this was not observed in patients who ultimately died. The multivariate logistic-regression model results indicated that lymphopenia that did not resolve after five days was a risk factor for death. Furthermore, we determined the CD4 and CD8 T cell counts in most of the patients included in our study. The results indicated a reduction in CD4 and CD8 T cell counts in about half of the patients during the early stage of 2009 H1N1 virus infection, which was similar to previous reports in China [7,32]. These findings indicated that lymphopenia was mostly caused by T cell reduction, in particular a reduction in CD4 T cells. We were unable to determine whether the low immunity was pre-existing or caused by H1N1 infection because we did not know the basic values for each patient. During the severe acute respiratory syndromes (SARS) epidemic, lymphopenia was considered to be caused by the virus infection but further studies are needed to investigate the precise host immune response to 2009 H1N1 influenza virus. These results suggest the physicians should pay close attention to patients who are infected with 2009 H1N1 influenza virus, who are also obese or have experienced long-term lymphopenia.

In the 1918-1919 influenza A pandemic, most deaths were attributed to concurrent bacterial infection [31]. A report into 2009 pandemic H1N1 influenza also indicated that 29% of the patients displayed bacterial coinfection, which might have contributed to the death rate in the current pandemic [33]. In our study, only 16% patients had positive blood or sputum cultures, and most of these pathogens could potentially be responsible ventilator-associated pneumonia. The majority of bacterial co-infections were drug-resistant bacteria such as *A. baumannii *and methicillin-resistant *S. aureus*, whereas non drug-resistant bacteria were found predominantly in other reports [33,34]. This result may reflect the abuse of antibiotics in China, and Chinese physicians should take measures to ensure protection against respiratory machine-related pneumonia, especially involving drug-resistant bacteria. To achieve this, physicians, nurses and patients should obey the rules of segregation and sterilization strictly and pay attention to bacteria contamination and the rational use of antibiotics. Few bacterial co-infections were detected in patients who did not have mechanical ventilation, which was consistent with most previous studies [14,26]. However, bacterial diagnostic tests were not performed for all patients, especially those who were not admitted to an ICU, and most patients received antibiotics close to the time of culture collection, which could have reduced the diagnostic sensitivity.

Although antiviral therapy is most beneficial when treatment is initiated within 48 hours after the onset of illness [35], a prospective cohort study of oseltamivir therapy in patients hospitalized with influenza infection indicated a reduction in mortality, even when such therapy was initiated more than 48 hours after illness onset [36]. Recent data from Thailand also showed that oseltamivir therapy was associated with survival in hospitalized patients with influenza pneumonia [37]. Under an Emergency Use Authorization, the FDA recently approved oseltamivir therapy for 2009 H1N1 infection even if it is initiated more than 48 hours after the onset of illness and also approved its use in children under the age of one year [35]. In our study, antiviral drugs were administered to all patients, but such therapy was not initiated within 48 hours of the onset of illness in all patients and there was no difference between surviving patients and those who died in the median number of days from the onset of illness to oseltamivir initiation. Therefore, we were unable to conclude whether or not antiviral therapy in critically ill patients led to better clinical outcomes. No patients undertook the test of the isolated 2009 H1N1 influenza A strains for oseltamivir resistance in our study, so whether oseltamivir resistance affected the outcomes of patients with 2009 H1N1 infection was unclear. Delayed initiation of antiviral therapy may have contributed to an increased severity of illness in our study.

Our study has several limitations. The patients we evaluated represented 30% of the total hospitalizations in Shenyang for 2009 H1N1 infection that were reported to the CDC during the surveillance period that ended in December, 2009. No children or pregnant women were included in our study. Participation in the study was voluntary and was therefore subject to reporting bias. We evaluated only patients with confirmed 2009 H1N1 infection, so the group may not be representative of all hospitalized patients as some may have gone undetected. All diagnostic testing was clinically driven, and tests were not obtained in a standardized fashion. Finally, despite the use of a standardized data-collection form, not all of the required information was collected for all of the patients and the sample size was small.

## Conclusions

During the evaluation period in Shenyang, 2009 H1N1 influenza caused severe illness requiring hospitalization. Thirty percent of the patients need an intensive care unit and fifteen percent died. Obesity and lymphopenia, which was not restored after five days of treatment, were factors associated with poor outcomes of 2009 H1N1 influenza infection. Early identification of pneumonia-susceptible patients at high risk of 2009 H1N1 influenza infection may aid clinicians in carrying out effective clinical management of this disease.

## List of abbreviations

CDC: centre for disease control; CRP: C reactive protein; LDH: lactic dehydrogenase; CK: creatine kinase; AST: glutamic-oxaloacetic transaminase; ALT: glutamic alanine aminotransferase; Alb: albumin; ICU: intensive care unit; BMI: Body Mass Index; COPD: chronic obstructive pulmanary disease; ARDS: acute respiratory distress syndrome; OR: odds ratio; CI: confidence interval; SARS: severe acute respiratory syndromes; WGOC: Working Group On Obesity In China; FDA: Food and Drug Administration.

## Competing interests

The authors declare that they have no competing interests.

## Authors' contributions

HZ, WC, XL, YW, YZ, BD, YW, WW and JK have made substantial contributions to acquisition of clinical data. PL and WC have made contribution to conception and design. WC has made contribution to analysis and interpretation of data. All authors read and approved the final manuscript.

## Author's information

Wei Cui is an associate professor in-residence in the Department of Infectious disease, the First affiliated hospital of China Medical University. Her research interests included the pathogenesis of infectious diseases, particularly the pathogenesis of fulminant hepatic failure and intestinal barrier disruption.

## Appendix

Normal values obtained from laboratory tests in adults:

Lymphocyte count: 0.8-4 ×10^9^/L; LDH: 313-618 U/L; CK: 30-135 U/L; AST: 13-33 U/L; ALT: 8-42 U/L; Alb: 35-50 g/L; CRP: 0-0.8 mg/mL; CD4 T cell: 410-1590/μL; CD8 T cell: 190-1140/μL; CD3 T cell: 690-2540/μL.

## Pre-publication history

The pre-publication history for this paper can be accessed here:

http://www.biomedcentral.com/1471-2334/10/145/prepub
